# Inhalations of Chloroform in Pneumonia

**Published:** 1853-10

**Authors:** 


*Inhalation of Chloroform in Pneumonia.*—The late journals of Germany publish more than 200 cases of pneumonia treated by inhalations of chloroform. Far from being contraindicated in pulmonary phlegmasia, as had been thought up to the present time, chloroform on the contrary would seem, according to these facts, to modify favorably the inflammatory process of the lung. From among the observations published, out of 193 cases treated by Drs. Wachner, Baumgartner and Schmit, only nine died. Of twenty-three cases reported by Dr. Wawentrapp, of Frankfort, nineteen were treated exclusively by chloroform, and only one died. Every two or three hours the patient is made to inhale the vapour from fifty drops of chloroform, during ten or fifteen minutes, so as never to let the effects reach a loss of consciousness. All the patients were of adult age, and the disease upon an average had reached the fifth day. In every case it was observed that the chloroform had a diaphoretic effect, which was sometimes produced by the first inhalation, and never failed to manifest itself on the third or fourth day. It gradually diminished the local pain, and caused it to disappear; it calmed the thoracic anxiety, brought back the respiration to its normal type, always appeased the cough, facilitated the expectoration in rendering it less abundant; and, lastly it reduced the febrile reaction and induced a refreshing sleep three or four days after the inhalations were commenced.—*Gazette des Hopitaux.*

